# Deciphering the pathogenic consequences of chromosomal aberrations in human genetic disease

**DOI:** 10.1186/s13039-014-0100-9

**Published:** 2014-12-19

**Authors:** Wigard P Kloosterman, Ron Hochstenbach

**Affiliations:** Department of Medical Genetics, Center for Molecular Medicine, University Medical Center Utrecht, Utrecht, 3508 AB The Netherlands; Department of Medical Genetics, Genome Diagnostics, Utrecht, 3508 AB The Netherlands

**Keywords:** Chromosomal aberration, Paired-end sequencing, Karyotyping, arrayCGH, Gene dosage, Gene fusion, Expression regulation, Expression profiling, Genome engineering

## Abstract

Chromosomal aberrations include translocations, deletions, duplications, inversions, aneuploidies and complex rearrangements. They underlie genetic disease in roughly 15% of patients with multiple congenital abnormalities and/or mental retardation (MCA/MR). In genetic diagnostics, the pathogenicity of chromosomal aberrations in these patients is typically assessed based on criteria such as phenotypic similarity to other patients with the same or overlapping aberration, absence in healthy individuals, *de novo* occurrence, and protein coding gene content. However, a thorough understanding of the molecular mechanisms that lead to MCA/MR as a result of chromosome aberrations is often lacking. Chromosome aberrations can affect one or more genes in a complex manner, such as by changing the regulation of gene expression, by disrupting exons, and by creating fusion genes. The precise delineation of breakpoints by whole-genome sequencing enables the construction of local genomic architecture and facilitates the prediction of the molecular determinants of the patient’s phenotype. Here, we review current methods for breakpoint identification and their impact on the interpretation of chromosome aberrations in patients with MCA/MR. In addition, we discuss opportunities to dissect disease mechanisms based on large-scale genomic technologies and studies in model organisms.

**Electronic supplementary material:**

The online version of this article (doi:10.1186/s13039-014-0100-9) contains supplementary material, which is available to authorized users.

## Introduction

Structural genomic variations (SVs) are generally regarded as genetic changes with a size larger than 50 bp [1]. SVs form a major source of common genetic variation in the human population and they primarily comprise deletions, (mobile element) insertions and tandem duplications [2]. Smaller structural variations occur much more frequently than larger ones. Furthermore, there is significant purifying selection against large and gene-disruptive copy number variations (CNVs), indicating their considerable phenotypic impact. The rates at which large CNVs (>100 kb) arise *de novo* in the general population have been conservatively estimated to be around 1.2×10^−2^ CNVs per germ line transmission [3]. This is relatively low when compared to the rates of base substitutions [4]-[6]. However, large CNVs and SVs have a high chance of affecting important genomic elements, which could lead to congenital disease. In line with this, large copy number changes are strongly enriched among patients with idiopathic multiple congenital abnormalities and/or mental retardation (MCA/MR) phenotypes compared to normal individuals [7]. In addition, *de novo* CNVs are found at higher frequency in MCA/MR patients (3.6×10^−2^ per germ line transmission) compared to control populations [8].

In spite of the clear association between large (*de novo*) CNVs and other SVs (collectively known as chromosomal aberrations) with MCA/MR phenotypes [9], precise characterization of molecular mechanisms that cause disease in individual patients is often unknown. This is largely a result of the complex effects of breakpoints on gene structure, function and expression. In the following sections we first provide an overview of current methods for diagnostic detection and interpretation of chromosomal aberrations in MCA/MR patients. Subsequently, the introduction and relevance of new methods for high-resolution dissection of breakpoints of chromosomal aberrations is discussed. Finally, we summarize the possible molecular consequences of chromosome aberrations for gene expression and function, and we discuss approaches for identification and unraveling the molecular determinants of congenital disease phenotypes.

## Review

### Diagnostics of chromosome aberrations in patients with MCA/MR

#### Current methods for detection of chromosome aberrations in genetic diagnosis of idiopathic MCA/MR

(Mosaic) aneuploidies and chromosomal rearrangements are a frequent cause of idiopathic MCA/MR. Starting in 1959 with the identification of trisomy-21 as the genetic basis of Down syndrome [10], microscopic observation of metaphase chromosomes has for several decades been the method of choice for detecting chromosome abnormalities in MCA/MR patients. This includes both karyotyping of banded chromosomes and, since the nineties of the last century, also fluorescence in situ hybridization (FISH). In consecutive, unselected MCA/MR patients karyotyping enables the detection of pathogenic chromosomal abnormalities in about 4% of cases (excluding Down syndrome), despite its limited resolution to about 5–10 Mb. The aberrations mainly include a single loss or gain of a chromosomal segment (>90% of cases) [11].

The application of targeted FISH to detect rearrangements that are beyond the resolution of chromosome banding, adds another ~4% of diagnosed MCA/MR patients [11]. Targeted FISH on metaphase chromosomes has been particularly successful in the diagnosis of recurrent microdeletions that are mediated by nonhomologous allelic recombination (NAHR) between flanking low copy repeats (LCRs), such as Velo-Cardio-Facial (VCF)/DiGeorge syndrome in 22q11.2, Williams-Beuren syndrome in 7q11.23 and Smith-Magenis syndrome in 17p11.2 (and many others, that were more recently discovered using microarrays [12], or computationally predicted [13]). These recurrent aberrations can be efficiently identified by targeted FISH if the patient’s phenotype has been recognized by a clinican (“phenotype-first” approach).

In contrast, most non-recurrent, sporadic chromosome rearrangements are characterized by breakpoints that are more or less randomly located, as they are typically driven by mechanisms of non-homologous repair such as nonhomologous end joining (NHEJ) and microhomology mediated break induced replication (MMBIR) [14]-[16]. Many of these private rearrangements may be associated with unique clinical phenotypes, requiring a “genotype-first” approach. The introduction of microarray-based aneuploidy detection has enabled such a “genotype-first”, discovery-based approach. With high-resolution microarray platforms, on which all protein-coding genes are addressed with multiple probes, the diagnostic yield in the population with MCA/MR referral is about 15% [11],[17]. Therefore, microarray-based aneuploidy detection has become the initial test in the laboratory work-up of patients with idiopathic MCA/MR [17]. Limitations of arrays include the inability to detect low-level chromosomal mosaicism (<7-10%) and balanced rearrangements. However, in diagnostics of idiopathic MCA/MR, karyotyping would add less than 1% of pathogenic cases to those detected by microarray, as shown by a survey of 36,325 consecutive MCA/MR cases [11].

#### Current methods for the assessment of the clinical relevance of chromosomal imbalances in idiopathic MCA/MR

Before CNVs could be precisely delineated using microarrays, traditional approaches for assessing whether an imbalance is causal for a patient’s clinical symptoms have been based on (i) the phenotypic resemblance to other patients with an identical or largely overlapping imbalance, (ii) the absence of the imbalance in a large number of healthy individuals, and (iii) the segregation of the CNV in the family, for example, *de novo* imbalances are considered to be likely pathogenic whereas those inherited from an apparently healthy parent are not. These criteria still hold true nowadays for the interpretation of CNVs [18]. However, the advent of the human genome sequence (and all its associated databases for genome annotation) has - together with the ability to precisely delineate imbalances using arrays - provided additional, sequence content-dependent features of CNVs. Most important, the presence of a gene in the imbalance with proven dosage effect or association with a known clinical disorder is considered as robust evidence for a pathogenic effect. Public databases such as ECARUCA, DECIPHER, ISCA, and PubMed (for comparison to other patients), and the Database of Genomic Variants (DGV) (for checking whether the CNV occurs in healthy individuals) are indispensible for the routine diagnostic workflow in the clinic [17]-[20].

Additional methods are being developed based on functional enrichment analysis of the genetic content of CNVs [21]. For example, CNVs are more likely to contribute to the patient’s phenotype if they contain genes with temporal and spatial patterns of expression that are in line with the phenotype seen in the patient, or if they contain genes with mouse phenotypes similar to symptoms in the patient. Furthermore, it has been shown that CNVs identified in patients with idiopathic MR are more likely to be pathogenic if they contain genes with mouse orthologues that cause abnormal neuron morphology or neurodegenerative disease when disrupted [22],[23]. More recently, it was shown that CNVs in patients with MR or schizophrenia are enriched in miRNA genes with brain related functions compared to common CNVs [24],[25].

### Sequencing of breakpoints from chromosomal aberrations to decipher rearrangement structure and identify affected genes

#### Detection of structural variation using next-generation sequencing

The introduction of next-generation DNA sequencing has accelerated the discovery of SVs in the human genome. In a landmark paper by Korbel *et al*., paired-end sequencing was introduced to identify more than thousand SVs in the human genome at unprecedented resolution [26]. Paired-end sequencing involves the mapping of pairs of sequence reads to the human reference genome [1]. These pairs are derived from the two ends of a single genomic DNA segment. A larger distance between the two reads that form a pair can be achieved by mate-pair sequencing [26],[27].

**Figure 1:**
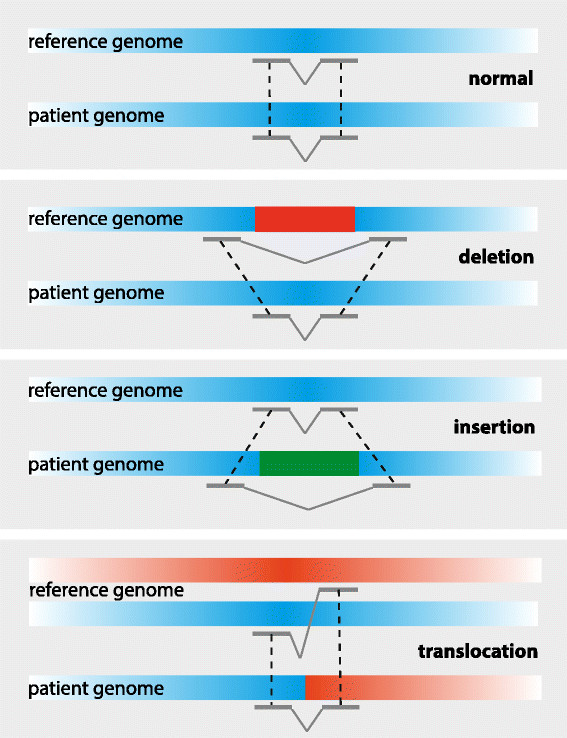
**Examples of read pair signatures that indicate the presence of different types of chromosomal aberrations.** Read pairs are indicated in grey. Read pair analysis uses information about the mapping position and orientation of sequence reads within a pair relative to each other.

Genomic breakpoints are detected following mapping of the read pairs to the reference genome. The mapping locations and orientations of the two reads will be concordant relative to each other if no breakpoint is present in between the two reads with respect to the reference genome. On the contrary, discordant mapping locations and orientations indicate a possible structural genomic change. Discordant read pair signatures that denote different types of structural variation are shown in Figure 1. Because the analysis of discordant reads is dependent on mapping of sequence reads in the vicinity of breakpoints, this approach is less feasible for recurrent chromosomal rearrangements.

Besides discordant read pair analysis, two other approaches are often used to detect structural genomic variation based on next-generation sequencing [1]. The analysis of the depth of sequence read coverage (DOC) is of great help to determine copy number status, while split read mapping gives insight into the precise breakpoint junction sequence.

#### The use of paired-end sequencing to fine-map rearrangement breakpoints in patients with MCA/MR

The first efforts to characterize genomic breakpoints of chromosomal aberrations in MCA/MR patients were performed by Chen *et al*. [28]. To limit sequencing costs, these authors used paired-end sequencing of derivative chromosomes isolated by flow sorting. Another strategy to limit sequencing costs and efforts concerns the sequence enrichment of breakpoint regions which have been previously established based on karyotyping or FISH studies [29],[30]. Such methods involve the design of capture probes within breakpoint regions and subsequent enrichment of these regions using on-array or in-solution enrichment of genomic libraries.

**Figure 2:**
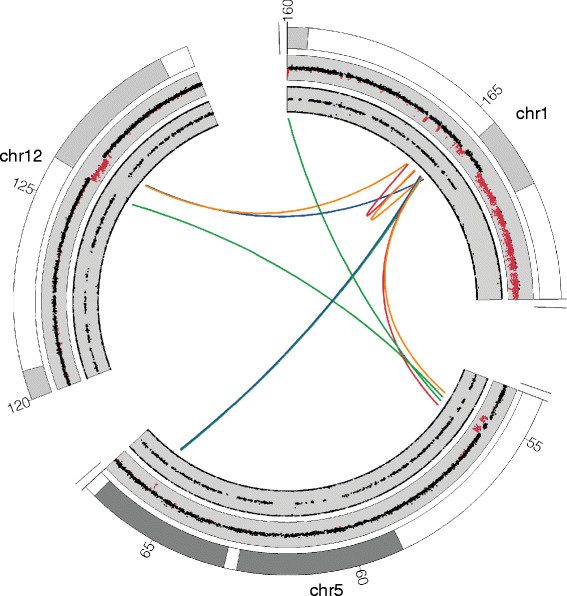
**Circos plot indicating** ***de novo*** **copy number changes and breakpoint junctions on chromosomes 1, 5 and 12 in a patient with severe congenital abnormalities.** The outer circle displays a partial chromosome ideogram (with numbers denoting megabase positions). The two grey inner circles display the log ratios and allele frequencies of the copy number profile, respectively, based on Illumina SNP-array analysis. The red dots indicate deletions. The colored lines in the middle part of the circle denote breakpoint junctions derived from mate-pair sequencing. The color indicates the junction orientation. Blue = tail-to-head; green = head-to-tail; red = head-to-head inverted; yellow = tail-to-tail inverted. This example illustrates that copy number profiling provides only a glimpse of the complexity of chromosomal aberrations.

With decreasing costs of next-generation sequencing, various studies have described the use of whole-genome sequencing to identify genomic breakpoints of balanced and unbalanced chromosomal rearrangements at nucleotide resolution [31]-[37]. Differences in approach in these studies mostly comprise the generation of genomic libraries with large (mate-pair) or short (paired-end) insert sizes. This has implications for the amount of sequencing reads that are needed to capture breakpoints: the smaller the insert-size of the library, the more sequencing reads are needed. The major conclusion from these studies is that underlying gene defects can be directly identified. In addition, the orientation of junction fragments provides a precise view of the rearrangement structure, which is impossible to reach by microarray or cytogenetic investigation only. This is particularly important for complex genomic rearrangements where multiple genomic segments have been rearranged, such as those caused by chromothripsis (Figure 2) [35]-[37]. In fact, sequencing has often revealed an unanticipated complexity of chromosomal aberrations [29],[32],[38], such as a high frequency of inversions associated with balanced chromosomal rearrangements [37]. The high resolution at which rearrangement breakpoints are mapped using next-generation sequencing requires new nomenclature for their clinical reporting [39].

#### Mapping disease genes using paired-end sequencing

Next generation sequencing of chromosomal breakpoints provides direct insight into the genomic elements – e.g. genes or regulatory DNA sequences - which are affected by the breakpoints. The precise definition of the genomic breakpoints and the local architecture of the chromosomal rearrangement is an essential first step towards the understanding of genetic disease mechanisms. For example, sequencing the breakpoints of a *de novo* translocation or inversion provides a direct view on the disrupted genes that may be relevant candidates genes for disease [29],[31],[33],[34],[40]-[45]. What is clearly hampering in most of these studies is the sporadic nature of the breakpoints in patients with a balanced rearrangement. Thus, it is difficult to establish a causal relationship between a gene and a clinical phenotype. Only in exceptional cases multiple translocation carriers were found, leading to firm associations between a gene and a phenotype [46]-[48]. An additional strategy to strengthen genotype-phenotype relationships involves the assessment of patients with copy number changes encompassing the same gene(s) as disrupted by the balanced rearrangement breakpoints [49]-[52]. The information derived from the sequencing of breakpoints from balanced translocations can also be of benefit to identify disease genes for microdeletion syndromes [52].

### The molecular consequences of chromosome aberrations

The effects of breakpoints or copy number changes can be versatile and multiple effects may result from a single chromosomal aberration. The following paragraphs highlight different molecular consequences of chromosomal aberrations observed in patients with congenital phenotypes.

#### Dosage effects

Genes that are completely encompassed by a CNV undergo a dosage change, which is expected to coincide with a change in mRNA expression level. This may have severe effects, because for a substantial amount of genes a single functional copy is insufficient for normal gene function [53]. The impact of CNVs on mRNA expression differences has been observed at the genome-wide level in human cell lines [54]. Copy number status correlates with mRNA expression but also genes outside of CNV intervals show altered expression [55],[56]. Combined analysis of CNV and transcriptome data from patients with autism spectrum disorder (ASD) revealed significant enrichment of dysregulated mRNA expression for genes within *de novo* CNVs when compared to common CNVs [57]. At the level of individual CNVs, studies have reported the effects of gene dosage on mRNA levels for e.g. VCF syndrome deletion, Williams Beuren syndrome deletion [58] and 16p11.2 deletion and duplication [59]. In the latter case, for virtually all genes spanned by the CNV interval, gene dosage correlated well with mRNA expression level.

To counteract the loss or gain of a chromosome (or chromosomal segment), a phenomenon known as dosage compensation can occur [60]. This effect may occur both at the level of gene transcription and translation [61]. Dosage compensation could explain the lack of correlation between copy number loss and mRNA expression level for some common CNVs in the human population [55]. Also, only 29% of genes on chromosome 21 in patients with Down syndrome show higher transcript levels, whereas the other 71% of genes show dosage compensation or variable expression among different patients [62]. A major hurdle to precisely define effects of CNVs on gene expression is the general variability in mRNA expression between different individuals. Therefore, a matched control experiment is often not available. A recent and very elegant study has overcome this limitation and reported a unique case: a monozygotic twin pair discordant for trisomy 21. Genome-wide differential expression patterns between the twins uncovered the presence of gene expression dysregulation domains (GEDDs), which contain either up- or downregulated genes [63].

#### Gene fusions

Gene fusions (also known as chimeric genes) can occur when two genomic segments – each containing (part of) a gene - join together and form a novel protein or a new promoter-gene fusion. Gene fusions are well studied in cancer, where they form active subjects of positive selection during tumor development [64]. Yet, in patients with MCA/MR the role of gene fusion is largely unclear. A few reports have characterized gene fusions associated with congenital disease phenotypes, often resulting from a balanced translocation. The gene fusions can be classified into several different classes. First, bona-fide in-frame gene fusions leading to novel, chimeric proteins can arise [65],[66]. In a patient with mental retardation, ataxia and atrophy of the brain, a *PAFAH1B3*-*CLK2* fusion was found, but a gain-of-function effect could not be observed [65]. The most likely explanation for the phenotype in this patient was the truncation (functional hemizygosity) of *PAFAH1B3*. Functional hemizygosity was also suggested for an in-frame *TNS3*-*FGFR1* gene fusion, which led to impaired FGFR1 function [67]. In other patients novel fusion proteins were observed, which consisted of part of a known protein coupled to a novel open reading frame that lacks homology to known proteins [68]-[70]. A striking negative effect on mitochondrial function was observed for novel *DISC1* fusions, which involve part of the DISC1 protein coupled to a novel peptide sequence, possibly accounting for psychiatric disease in a large family [71]. A second possibility entails the fusion of a promoter to the coding regions from another gene. However, this has not yet been described for MCA/MR patients.

Two large-scale studies have used CNV datasets from patients with autism spectrum disorders (ASD) and schizophrenia, respectively [72],[73]. An increased frequency of brain-expressed in-frame gene fusions was found in schizophrenia cases, but not in patients with ASD. Two fusion genes in the schizophrenia cohort displayed different subcellular locations compared to their parental genes, suggesting a role in disease etiology.

In all cases described above, the observed gene fusions were unique to a single patient, precluding the establishment of a causal relationship to the disease phenotype. Clearly, determining the precise role of gene fusion in congenital disease requires detection of breakpoints at nucleotide resolution, identification of recurrent fusions and in-depth functional characterization of individual cases.

#### Deregulation of gene expression through dislocation and disruption of non-coding elements

In addition to direct disruption of coding sequences through a CNV or SV breakpoint, chromosomal rearrangements may also affect regulatory elements, which in turn alter gene transcription. In fact, the altered regulation caused by disruption of regulatory elements may result in subtler gene expression changes - such as during specific developmental stages or in specific tissues – and not be a full loss of function (for review see: [74]). This leads to different phenotypes depending on the type of regulatory element that is affected or the severity of the effect. For example, Pierre Robin sequence is caused by chromosomal aberrations affecting noncoding enhancer elements upstream of the *SOX9* gene [75],[76]. The deregulation of *SOX9* is determining the final phenotypic outcome: translocation breakpoints close to *SOX9* typically result in campomelic dysplasia, whereas those further upstream cause acampomelic campomelic dysplasia [77]. Overall, the skeletal phenotypes tend to be less severe with increased distance between breakpoints and *SOX9*[77]. Besides distance to a gene, a second factor of importance for the molecular consequences of the disruption of non-coding elements by chromosomal aberrations is the type of element that is disrupted [74]. Loss of enhancer function in the case of Pierre Robin sequence, causes reduced *SOX9* expression. In contrast, deletion of a small genomic silencer element just upstream of the delta-globin gene, provokes fetal hemoglobin expression [78]. Another mechanism concerns the relocation of enhancer elements relative to coding regions, causing them to control activity of new genes. Such a position effect has been observed in a patient with an inversion that dislocates the *SHH* gene and places it under the control of a highly active limb bud enhancer element [79]. Systematic survey of deletions from the DECIPHER database has revealed that 11.8% of patient phenotypes could best be explained by enhancer adoption rather than mere dosage effects of genes within the deletion interval [80]. These and other effects of chromosomal aberrations on noncoding DNA elements have only recently been recognized, but are essential for diagnostic interpretation [74].

#### Unmasking of recessive mutations by chromosomal deletions

Whenever a deletion occurs, any recessive mutation on the non-deleted homologous chromosome would become unmasked, and could contribute to the phenotype. Indeed, there is anecdotal evidence for the unmasking of a mutated, pathogenic allele on the homologous, non-deleted chromosome [81]. This phenomenon may be one of the explanations for the phenotypic variability between unrelated patients with identical deletions. The best studied case is the 3 Mb 22q11.21 deletion mediated by NAHR between LCRs A and D that occurs *de novo* in about 90% of patients with DiGeorge/VCF syndrome [82]. For example, mutations affecting the *GP1BB* gene have been identified in several patients with features of both DiGeorge/VCF syndrome and Bernard-Soulnier syndrome, a rare autosomal recessive bleeding disorder [83],[84]. Other examples include the unmasking of a *SCARF2* splice site mutation in a patient with DiGeorge/VCF syndrome and additional features of Van den Ende-Gupta syndrome, such as arachnocamptodactily and typical facial anomalies such as blepharophimosis, beaked nose and malar hypoplasia [85], and the unmasking of *SNAP29* frameshift mutations in two patients who also had features of CEDNIK syndrome (cerebral dysgenesis, neuropathy, ichthyosis and keratoderma) [86]. It should be noted that these patients were investigated in-depth because they displayed features of more than one syndrome or condition. This suggests that such patients are rare, exceptional cases. This is supported by a the lack of evidence for the unmasking of selected candidate genes in other recurrent deletion syndromes, such as the 1q21.1 deletion associated with thrombocytopenia-absent radius syndrome [87], the 16p13.11 deletion associated with MR and autism [88], and the 16p11.2 deletion associated with autism [89].

There are also some examples of inherited deletions that unmask a recessive pathogenic allele [81]. In theory, this mechanism could explain the phenotypic difference when there are affected and unaffected carriers of the deletion in a family. This situation occurs, for example, when there is an affected child with the deletion and an unaffected, apparently healthy carrier parent. This theory was tested in a cohort of 20 familial deletion cases [81]. Only in one case a 16.0-31.7 kb deletion containing only the *HSBP1* gene in 16q23.3 inherited from one parent was unmasked by a 2.2 Mb deletion inherited from the other parent.

In summary, the unmasking of pathogenic mutations may be a rare phenomenon, both in recurrent, *de novo* deletions and in familial deletions. It is possible, however, that this mechanism operates in more subtle ways, i.e. by unmasking sequence variants that occur in the general population. For example, the risk of developing schizophrenia and other neuropsychiatric conditions in 22q11.2 deletion patients is dependent on DNA-sequence polymorphisms on the intact chromosome that affect the function or expression of genes such as *COMT*, *PIK4CA*, and *GNB1L*[90]. There is a clear need for systematic studies of genetic variation in the non-deleted genes, including miRNA genes [91] in large cohorts to assess the contribution of unmasking to our understanding of the phenotypic variation between patients with identical deletions.

### Strategies for functional characterization of chromosome aberrations

Extensive functional characterization of chromosomal aberrations is needed to identify the critical molecular determinants of a patient’s phenotype. It may well be that some of the effects of breakpoints on gene function are not harmful and can be regarded as passenger events, while other effects are predominantly driving disease. To discriminate between passenger and driver effects a variety of approaches can be applied, ranging from large-scale genomics to functional studies in model organisms.

#### Large-scale genomics technologies

The molecular effects of structural genomic variations may expand far beyond the genomic region that is directly affected by breakpoints or copy number change. Genome-wide molecular profiling is helpful to pinpoint consequences of SVs in an unbiased fashion. For example, mRNA expression analysis has been performed in families including patients with ASD to identify autism-susceptibility genes associated with CNVs [57]. One important limitation of mRNA expression studies is the tissue type chosen for investigation. In most cases blood is used as a proxy for expression in the brain. A second limitation is the lack of a proper control dataset. Healthy family members can be used as a control, but differences in genetic background contribute already substantially to gene-expression differences. Indeed, the study by Luo *et al*. [57] showed that significantly misexpressed genes are not restricted to probands, but are equally prevalent in unaffected siblings. However, gene ontology analysis revealed an enrichment of misexpressed genes in neural-related pathways and misexpressed genes are localized within pathogenic CNV intervals. Finally, the effects of CNVs on gene expression may not be extending throughout lifetime, as studies in mouse revealed temporary compensatory loops of brain-expressed genes at specific developmental time-points [92].

Application of mRNA sequencing to a cohort of patients with reciprocal 16p11.2 duplication or deletion revealed most prominent changes within CNV intervals, but also several effects in *cis* and *trans*[59]. These effects were associated with changes in long-range physical interactions identified by Hi-C chromosome conformation capture technology. Chromosome conformation capture methods provide insight into nuclear organization and identify chromosomal regions that physically interact with each other [93]. SVs can change the organization of chromatin and thereby affect transcription of nearby genes, as was recently shown for deletion of the Williams-Beuren Syndrome region [94].

Finally, the impact of chromosomal rearrangements and SVs on regulatory regions in the genome can be identified by the analysis of histone modifications that mark active promoters and enhancers, such as H3K4Me3 and H3K27Ac, respectively [95]. Effects of SVs on histone modifications have hardly been studied, but may provide important information on global changes in gene regulation [94].

#### Engineering rearrangements using genome editing technology

Genome engineering with programmable nucleases provides an extremely powerful approach for introducing specific mutations into human cells or model organisms [96]. These systems are often used to enable gene disruption by generating a nuclease-mediated double-stranded break, leading to frame-shifts or other disruptive mutations. However, expression of two site-specific nucleases allows engineering of chromosomal rearrangements, such as deletions, inversions and translocations [97]-[100]. For example, cancer–related translocations and inversions leading to fusion genes were mimicked in human cells [99]. These technological breakthroughs provide unique opportunities to engineer specific chromosomal rearrangements that occur in patients with MCA/MR. Such an approach circumvents measurement noise resulting from differences in genetic background, which has, for example, hampered the detection of gene-expression changes in case–control studies for various microdeletion or microduplication syndromes [57],[59],[101],[102]. Large-scale genomics analyses or specific functional assays on cells with engineered rearrangements and controls will reveal detailed insight into genome-wide effects. Furthermore, introduction of chromosomal aberrations in induced pluripotent stem (iPS) cell lines [103] or primary neuronal cell cultures [104] allows modeling facets of human disease, such as neuronal differentiation. Finally, the effects of chromosomal rearrangements on non-coding elements in the genome can best be studied by deleting an entire locus by cutting with two site-specific nucleases.

#### Understanding functional consequences of chromosomal aberrations in model organisms

Functional characterization of chromosomal aberrations in model organisms can be of high value to understand disease mechanisms or strengthen genotype-phenotype associations. Several mouse models have been generated to investigate recurrent microdeletion and microduplication syndromes. An elegant study described mouse models with CNVs that affect regions syntenic to human chromosomal band 17p11.2 [105]. Deletions and duplications in 17p11.2 lead to Smith-Magenis and Potocki-Lupski syndrome, respectively. These mouse models recapitulate much of the phenotypes observed in human subjects. The dosage of the *RAI1* gene appears to be most critical to these phenotypes [106],[107]. The effects of the CNVs were further studied in several mouse tissues revealing that many expression changes map to the engineered CNV intervals. Strikingly, restoring copy number by generating a mouse strain with a deletion and a duplication chromosome did not completely rescue some neurobehavioral phenotypes. Thus, a disturbance of local genomic architecture also plays a role in disease, in addition to gene dosage effects. Similar studies in mouse models of the 16p11.2 deletion and duplication were instrumental to map the changes in expression networks that account for the ASD phenotypes that are associated with these CNVs [59].

Zebrafish (*Danio rerio*) form an alternative to mouse models and are exceptionally suited for studying early embryonic development. Furthermore, zebrafish embryos can be easily manipulated allowing quick evaluation of many genes and mutations [108]. Overexpression in zebrafish embryos of genes within the 16p11.2 CNV interval revealed *KCTD13* as the major driver of the microcephaly phenotype seen in patients with the 16p11.2 duplications [109]. On the contrary, knockdown of *KCTD13* resulted in macrocephaly, which is typical for 16p11.2 deletion carriers. These results indicate that a single gene may form the primary driver of disease even though the entire 16p11.2 CNV region encompasses 29 genes. Thus, screening of large numbers of genes that are affected by dosage changes or breakpoints of chromosomal aberrations is extremely informative to understand disease mechanisms. A similar approach of functional testing in zebrafish was used to dissect the phenotypic determinants of the 8q24.3 copy number variant [110]. This revealed that two genes, *SCRIB* and *PUF60*, are primary drivers of disease. These selected examples demonstrate that zebrafish appears a very powerful model system to systematically dissect the functional consequences of chromosomal aberrations.

## Conclusions

### The genetics clinic of the future

The resolution, speed and breadth at which a patient’s genome can be scanned for pathogenic changes have increased dramatically over the last decade. The use of microarrays as the initial genetic test is likely to change in the coming years. By the application of whole exome sequencing, pathogenic gains and losses can be identified on a genome-wide scale, in parallel to pathogenic mutations in (protein coding) genes [111]-[113]. Although whole exome sequencing has lead to rapid discovery of disease genes, trends are shifting towards whole genome sequencing, as this is now an affordable method to identify all classes of genetic changes across the entire genome. In a recent study, whole genome sequencing was used to map *de novo* genetic changes in a group of patients with severe intellectual disability, which had not received a diagnosis based on previous CNV profiling and exome sequencing [114]. In this cohort additional mutations and CNVs were identified in coding regions, enabling a genetic diagnosis in 20 out of 50 patients.

In the genetics clinic of the future, whole genome sequencing to detect pathogenic SVs and CNVs will become a routine analysis. The application of microscopy will then be limited to the determination of the structure of chromosomal rearrangements, such as, for example, the discrimination between trisomy-21 and Robertsonian translocations involving chromosome 21 in Down syndrome, as in 1959. Also the detection of low-level mosaicism for aneuploidy of entire or rearranged chromosomes may remain dependent on microscopy in the near future. A suspicion of mosaicism should be raised for patients with normal NGS findings who display asymmetric body features, and “deep karyotyping” of multiple tissues may be required to arrive at a clinical diagnosis [115].

The major challenge will be to address the functional consequences of pathogenic variants by large-scale genomic approaches and systematic studies in model systems. These efforts are needed to fully understand the molecular effects of chromosomal aberrations and their role in disease etiology. Detailed insight in disease mechanisms will improve diagnostics, allow for pre-symptomatic screening for complications, support short and long-term prognosis and design of targeted and personalized medical treatment strategies.

## Authors’ original submitted files for images

Below are the links to the authors’ original submitted files for images. Authors’ original file for figure 1 Authors’ original file for figure 2

## Abbreviations

ASD: Autism spectrum disorder
CEDNIK: Cerebral dysgenesis, neuropathy, ichthyosis and keratoderma
CGH: Comparative genomic hybridization
CNV: Copy number variation
DECIPHER: DatabasE of Chromosomal Imbalance and Phenotype in Humans using Ensembl Resources
DGV: Database of Genomic Variants
DOC: Depth of coverage
ECARUCA: European Cytogeneticists Association Register of Unbalanced Chromosome Aberrations
FISH: Fluorescence in situ hybridization
GEDD: Gene expression dysregulation domains
IPS: Induced pluripotent stem
ISCA: The International Standards for Cytogenomic Arrays Consortium
LCR: Low copy repeat
MCA/MR: Multiple congenital abnormalities and/or mental retardation
MMBIR: Microhomology-mediated break-induced replication
MR: Mental retardation
NAHR: Non-allelic homologous recombination
NHEJ: Nonhomologous end-joining
SNP: Single nucleotide polymorphism
SV: Structural variation
VCF: Velo cardio facial
