# Supplementary figures and images for: Deciphering the pathogenic consequences of chromosomal aberrations in human genetic disease
Source: Mol Cytogenet. 2014 Dec 19;7:100. doi: 10.1186/s13039-014-0100-9 (PMC4299681; doi:10.1186/s13039-014-0100-9)

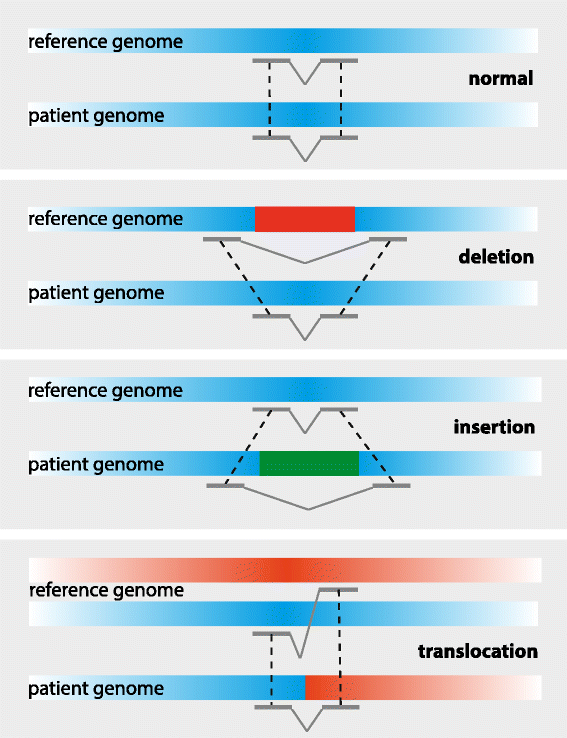

Supplement: Supplementary file 1 — Authors’ original file for figure 1 [file 13039_2014_100_MOESM1_ESM.gif]

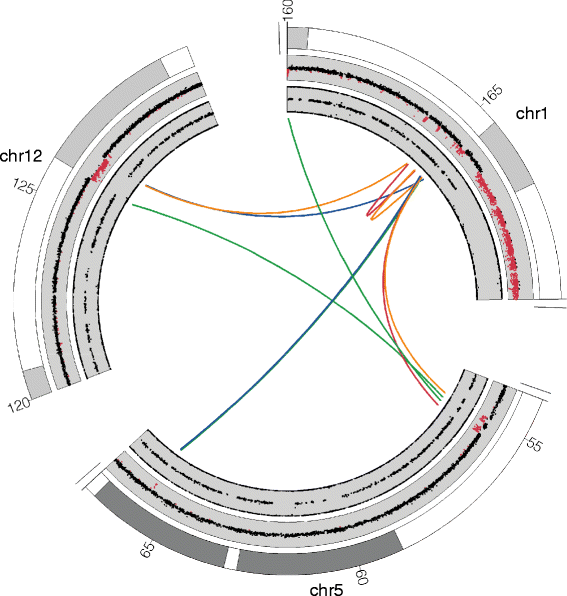

Supplement: Supplementary file 2 — Authors’ original file for figure 2 [file 13039_2014_100_MOESM2_ESM.gif]
